# On the Catalytic Effect of Water in the Intramolecular Diels–Alder Reaction of Quinone Systems: A Theoretical Study

**DOI:** 10.3390/molecules171113687

**Published:** 2012-11-20

**Authors:** Jorge Soto-Delgado, Arie Aizman, Renato Contreras, Luis R. Domingo

**Affiliations:** 1 Departamento de Química, Facultad de Ciencias, Universidad de Chile, Santiago, Casilla 653, Chile; 2 Departamento de Química, Universidad Técnica Federico Santa María, Valparaíso, Casilla 110-V, Chile; 3 Departamento de Química Orgánica, Universidad de Valencia, 46100, Valencia, Spain

**Keywords:** intramolecular Diels–Alder reactions, polar Diels–Alder reactions, water catalysis, DFT reactivity indices, local reactivity difference index

## Abstract

The mechanism of the intramolecular Diels–Alder (IMDA) reaction of benzoquinone **1**, in the absence and in the presence of three water molecules, **1w**, has been studied by means of density functional theory (DFT) methods, using the M05-2X and B3LYP functionals for exploration of the potential energy surface (PES). The energy and geometrical results obtained are complemented with a population analysis using the NBO method, and an analysis based on the global, local and group electrophilicity and nucleophilicity indices. Both implicit and explicit solvation emphasize the increase of the polarity of the reaction and the reduction of activation free energies associated with the transition states (TSs) of this IMDA process. These results are reinforced by the analysis of the reactivity indices derived from the conceptual DFT, which show that the increase of the electrophilicity of the quinone framework by the hydrogen-bond formation correctly explains the high polar character of this intramolecular process. Large polarization at the TSs promoted by hydrogen-bonds and implicit solvation by water together with a high electrophilicity-nucleophilicity difference consistently explains the catalytic effects of water molecules.

## 1. Introduction

**Scheme 1:**
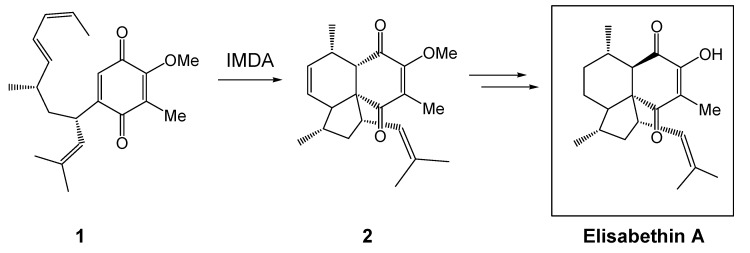
Synthesis of diterpenoid elisabethin A via an IMDA reaction.

The intramolecular Diels–Alder (IMDA) reaction is a powerful tool frequently employed in organic synthesis due to its versatility in the construction of fused cycles in only one synthetic step and also due to its remarkable stereoselectivity. These aspects have been used in a large number of processes that include numerous total synthesis of natural products [1]. On the other hand, the use of quinones as the dienophile component in Diels–Alder (DA) reactions provides access to a range of structures that are part of the fundamental skeleton of many natural products and biologically active molecules [2]. In this context, the marine diterpenoid elisabethin A, isolated from *Pseudopterogorgia elisabethae* [3,4], is an interesting target in organic synthesis due to its complex molecular structure and high functionalization. Heckrodt and Mulzer reported the first total synthesis of elisabethin A via an IMDA reaction (see Scheme 1) [5]. This reaction occurs with high stereoselectivity, but the major interest of this cycloaddition lies in its unusual reaction conditions: aqueous medium and room temperature.

Theoretical studies devoted to elucidate the role of the solvent in the acceleration of the reaction and also in its stereoselectivity have been reported previously [6]. Catalysis by water in quinone systems is well documented. Experimental data [7,8,9] suggest that the factors responsible for the acceleration of the IMDA process involve “enhanced hydrogen bonding” between the solvent and the transition state structure (TS) relative to the initial state of the reaction. Additionally, “enforced hydrophobic interactions” on the reactant hydrophobic surface area decrease during the activation process.

Theoretical investigations showed the separation and quantification of the relative terms that contribute to the enhanced rates using an explicit treatment of solvent employing QM/MM simulations [10,11]. The results reported from this hybrid methodology revealed a distance between the proton and the acceptor atom of about 2–2.5 Å. This interaction, although weak, is sufficient to polarize the carbonyl group which in turn produces an enhanced hydrogen bond (HB) at the TS. Each interaction is 1–2 kcal/mol more favourable per water molecule incorporated. On the other hand, Kong and Evanseck [12] studied the intermolecular DA reaction between butadiene and acrolein in aqueous phase using the B3LYP/6-31G(d) level of theory together with the polarizable continuum model (PCM). The authors showed that the microsolvation effect of the explicit water explains the decrease in the activation barriers and the *endo/exo* selectivity due to an electronic polarization of the carbonyl group at the TS [12].

The recent development of meta-GGA functionals, such as the M05-2X [13] or M06-2X [14], and their use in the TS geometry optimization and in Gibbs energy computations provide an accurate prediction for chemical reaction barriers to furnish an excellent representation of non-covalent interactions. In addition, the use of the hybrid B3LYP [15,16] functional in the geometry optimization step, followed by a single point Gibbs energy evaluation using the M05-2X [17] and M06-2X [18] functionals provides accurate predictions of activation free energies, and has been successfully used to study several mechanism in DA [19,20] and IMDA [21,22] reactions.

The purpose of this work is to contribute to a better understanding of the reaction mechanism in IMDA reactions in water and to characterize the possible effects of HB interactions in the catalytic process. The first part is a complete analysis of the potential energy surface (PES) for this reaction. The characterization of the TSs is performed by incorporating explicit water molecules. The study is completed with a natural bond orbital (NBO) population analysis and a reactivity index analysis based on a group electrophilicity variation [23,24] along the reaction coordinate.

## 2. Computational Details 

All structures were optimized using the M05-2X/6-31G(d) and B3LYP/6-31G(d) level of theory using the Gaussian 03 suite of programs [25]. The stationary points were characterized by frequency calculations in order to verify that the TSs had one and only one imaginary frequency. The intrinsic reaction coordinate (IRC) paths were traced to check the energy profiles connecting each TS to the two associated minima, the reactant and the product of the reaction. Implicit solvent effects of water were evaluated by performing single-point energy calculations at the gas-phase stationary points involved in the reaction using the PCM [26] with an integral equation formalism variant (IEFPCM) with UFF radii [27]. Thermodynamic data were calculated with standard statistical thermodynamics at 298.15 K and 1 atm [28].

Natural population analysis (NPA) was used to evaluate changes in the hyperconjugation and polarization upon formation of the hydrogen-bonded complex in reactants and TSs using the M05-2X/6-31G(d) level of theory. NPA analysis also assesses the CT patterns at the TSs. Visualization and figures were generated by *PyMOL* software [29].

The reactivity indices based on conceptual DFT were evaluated at the B3LYP/6-31G(d) optimized geometries of **1** and **1w**. The global electrophilicity index, *ω* [30], which measures the stabilization in energy when the system acquires an additional *ΔΝ* from the environment is given by the following simple expression: *ω = μ^2^/2η*, where μ is electronic chemical potential and η is the chemical hardness. This index has been used to classify the dienes and dienophiles currently used in DA reactions within a unique scale of electrophilicity [31]. Both the electronic chemical potential μ and chemical hardness η may be further approached in terms of the one electron energies of the frontier molecular orbital HOMO and LUMO, *ε_H_* and *ε_L_*, using the expressions μ ≈ (ε_Η_ + ε_L_)/2 and η ≈ (ε_L_ − ε_H_). In addition, the nucleophilicity index is defined [32,33] as *N* = ε_HOMO_ – ε_HOMO(TCE)_ where ε_HOMO_ is the HOMO energy of the nucleophile and ε_HOMO(TCE)_ corresponds to the HOMO energy of the tetracyanoethylene (TCE) taken as reference.

Recently, we have proposed the local reactivity difference index R_k_, [34] which is able to characterize the local electrophilic and/or nucleophilic activation within an organic molecule. The R_k_ index is defined as [34]:

if (1 < ω_k_/N_k_ < 2) or (1 < N_k_/ω_k_ < 2)

then R_k_ ≈ (ω_k_ + N_k_)/2     ⇒ ambiphylic (R_k_ = ±n.nn)

else R_k_ ≈ (ω_k_ − N_k_)

   where R_k_ > 0     ⇒ electrophilic (R_k_ = +n.nn) 

   and R_k_ < 0      ⇒ nucleophilic (R_k_ = −n.nn) 

if |R_k_| < 0.1, then R_k_ = 0

ω_k_ and N_k_ are the local electrophilicity [35] and nucleophilicity [36] indices, respectively, defined as:

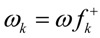

and:

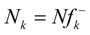

where *f_k_*^+^ and *f_k_*^−^ are the Fukui functions for electrophilic and nucleophilic attacks, respectively [37]. The Fukui functions can be obtained from single point calculations at the optimized structures of the ground state of molecules by a method described elsewhere [38].

In the R_k_ index, the sign (+, −, ±) indicates the electrophilic or/and nucleophilic character of the centre k, while the magnitude n.nn provides a measure of the local activation. For a molecule, the R_k_ molecular map of reactivity (RMMR) represents all local R_k_ indices, giving a general idea of its reactivity in polar processes

Recently we have introduced the fragment nucleophilicity and electrophilicity indices. They are defined as follows [23]: 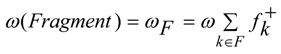

and:

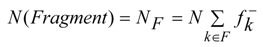

where F = diene (D) or the dienophile (Dp). 

The direction of the electronic flow within an IMDA reaction may be determined by the following dual indices:

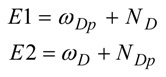


Thus, if E1 > E2 the process is expected to display a D to Dp electronic flow, whereas if E1 < E2 the process will be characterized by a Dp to D electronic flow. Note that for those cases where E1 ≈ E2, the model predicts that the IMDA process will follow a non-polar channel with negligible CT at the TS. The dual indices are used here to determine the electronic flow, which is associated with the D and Dp moieties. However, this qualitative model is not sufficient to describe the complete CT along the reaction coordinate. For this purpose we have developed a regional electrophilicity which is defined as: 
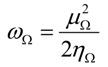

where Ω = D or Dp. This model of regional electrophilicity uses the inequality chemical potential principle [39]. The condensation process is however a little bit different because in the present case, the molecular orbital used are those centred at each molecular fragment [24].

## 3. Results and Discussion

**Scheme 2:**
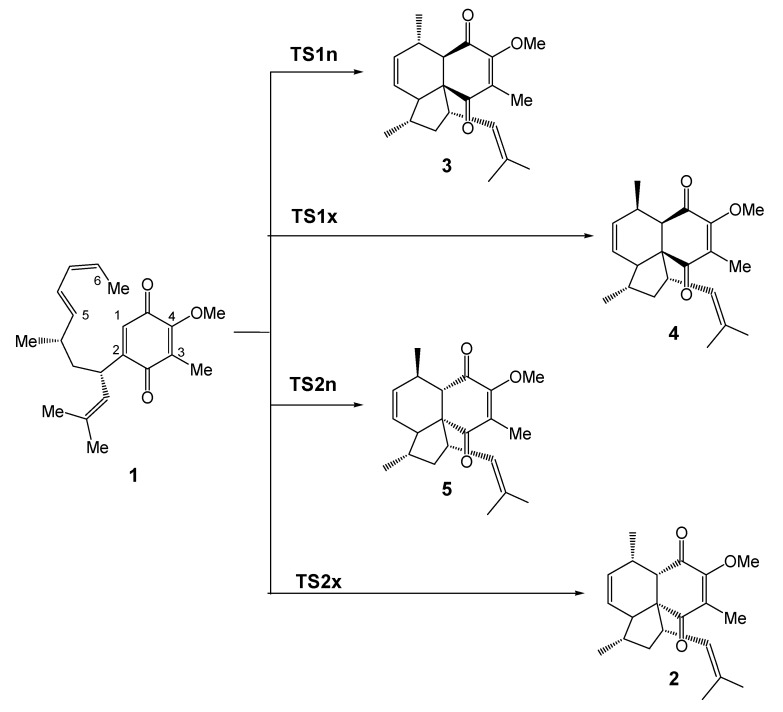
Competitive reaction channels associated with the IMDA of **1**.

*Energy and geometry aspects:* The IMDA reaction of quinone **1** can take place along four reactive channels resulting from the *endo* and *exo* stereoisomeric approach modes of the diene framework near the benzoquinone oxygens, and with the two stereoisomeric attacks on the two prochiral faces of the benzoquinone system. An exhaustive exploration of the PES for these IMDA reactions indicates that a one-step mechanism may be operative. Consequently, one TS and the corresponding [4+2] cycloadduct (CA) were located and characterized for each of the four stereoisomeric channels (see Scheme 2). Quinone **1** can be found in several conformations due to the free bond rotation of the appended chain, being in an energy range below 5 kcal/mol. For energy reference the most favourable extended conformation was selected.

Comparison of the gas-phase activation energies obtained with both functionals shows greater differences (see Table S1 in Supporting Information). B3LYP activation energies are between 7.7 and 11.0 kcal/mol higher that the M05-2X, while the IMDA reactions are between 15.2 and 23.7 kcal/mol lesser exothermic. However, as indicated in the introduction, M05-2X thermodynamic calculations on the optimized B3LYP geometries yield similar relative free energies than those obtained on the optimized M05-2X geometries (see Table S2 in Supporting Information).

**Figure 1:**
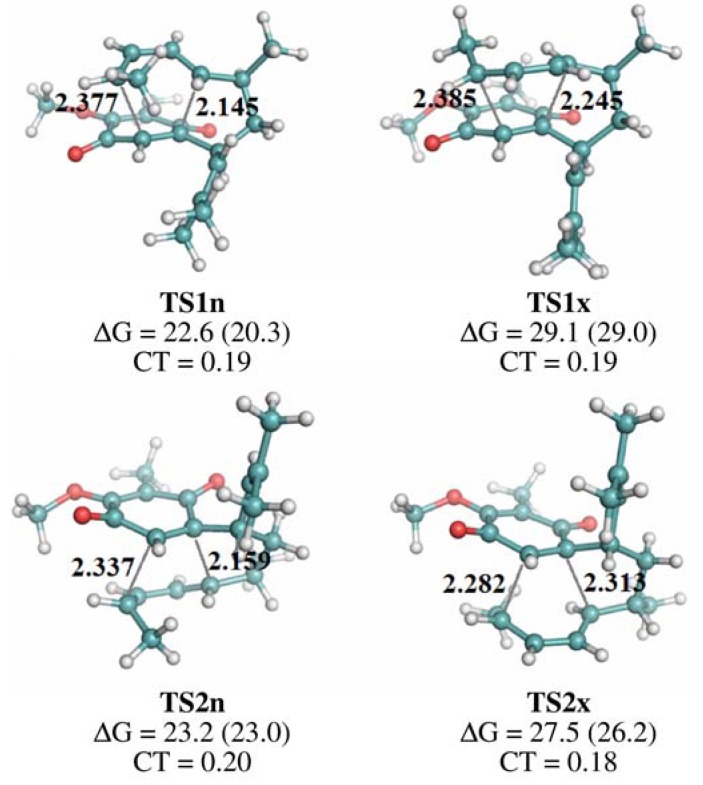
M05-2X/6-31G(d) Geometries of TSs for the IMDA reactions of quinone **1**. Bond distances in Å units. Activation free energies (ΔG, relative to **1**; in parentheses PCM calculations) in kcal/mol and CT in *e* units.

The gas phase M05-2X/6-31G(d) activation free energies associated with the IMDA reactions of quinone **1** are 22.6 kcal/mol for **TS1n**, 29.1 kcal/mol for **TS1x**, 23.2 kcal/mol for **TS2n** and 27.5 kcal/mol for **TS2x**. These results indicate that in gas phase, the reaction shows *endo* selectivity along the two diasterotopic faces. These IMDA reactions are exergonic in the range from −25.0 to −32.0 kcal/mol. Inclusion of solvent effects modelled by the PCM method produces a slight decrease of the activation free energies; 2.3 kcal/mol for most favored *endo* **TS1n** (see Figure 1). The M05-2X/6-31G(d) geometries, including the lengths of the two forming bonds and relative free energies of the TSs of the IMDA reactions of quinone **1**, are shown in Figure 1.

The extent of the asynchronicity of bond-formation can be measured by means of the difference between bond lengths of the two σ bonds that are being formed in the reaction, *i.e.*, Δd = d(C1-C6) − d(C2-C5). The asynchronicity at the TSs is: 0.23 at **TS1n**, 0.14 at **TS1x**, 0.18 at **TS2n** and −0.03 at **TS2x**. These rather low values indicate the low asynchronicity in these intramolecular processes. Explicit solvation increases the asynchronicity at the more favorable *endo* TSs.

NPA analysis provides information about the CT along these cycloadditions. The CT at the TSs is: 0.19*e* at **TS1n** and **TS1x**, 0.20*e* at **TS2n** and 0.18*e* at **TS2x**, thereby indicating that the TSs have some polar character.

**Figure 2:**
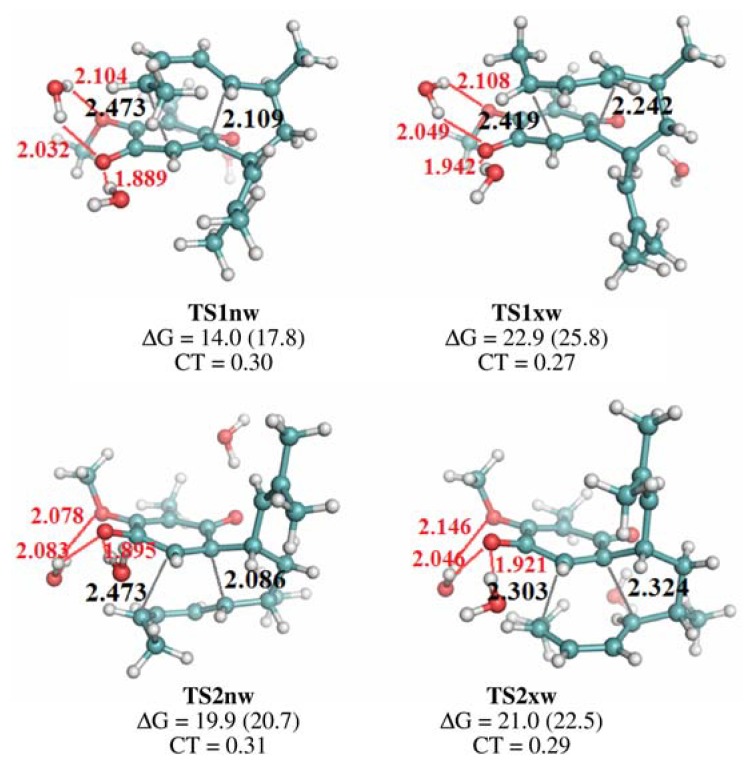
M05-2X/6-31G(d) Geometries of TSs for the IMDA reactions of **1w**. Bond distances in Å units. Activation free energies (ΔG, relative to **1w**; in parentheses PCM calculations) in kcal/mol and CT in *e* units.

Explicit solvation of quinone **1** with three water molecules decreases the gas phase activation free energies associated with the IMDA reactions of **1w** by 3.3 to 8.6 kcal/mol with respect to the IMDA reactions of quinone **1**. However, the further implicit solvation evaluated with the PCM method increases gas phase free energies by *ca.* 4.0 kcal/mol, due to a larger solvation of **1w** than TSs. Thus, the M05-2X/6-31G(d) activation free energies associated with the IMDA reactions of quinone **1w** in water are 17.8 kcal/mol for **TS1nw**, 25.8 kcal/mol for **TS1xw**, 20.7 kcal/mol for **TS2nw** and 22.5 kcal/mol for **TS2xw** (see Figure 2). These IMDA reactions are exergonic in the range from −27.9 to −33.5 kcal/mol.

The net water effects cause the *endo* approaches to be 8.0 (**TS1nw**) and 1.8 (**TS2nw**) kcal/mol more favourable than the *exo* ones. In addition, along the *endo* channels, **TS1nw** is located 2.9 kcal/mol below **TS2nw**. Thus, the IMDA reaction of **1w** in water presents complete *endo* selectivity, **TS1nw** being 8.0 kcal/mol lower in free energy than **TS1xw**, and high facial selectivity, **TS1nw** being 2.9 kcal/mol lower in free energy than **TS2nw**, yielding CA **3**. Similar *endo* and facial selectivities are found in the IMDA reaction of **1** (see Figure 1). On the other hand, the activation free energy associated with **TS1nw** in water is 4.8 kcal/mol lower in energy than that associated with **TS1n**. Therefore, both implicit and explicit water effects accelerate the IMDA reaction of **1**, but do not modify the *endo* and facial selectivities. This behavior can be understood considering that selectivities in IMDA reactions depend more on the strain energy associated with the intramolecular process than electronic effects, which control intermolecular processes. Note that the intermolecular DA reactions of methyl substituted 1,3-butadienes with 2-acetyl-1,4-benzoquinone, *endo* TSs are favored by 3.8 kcal/mol when compared with *exo* ones [40].

*Endo* CA **3**, which is formed by the nucleophilic attack of the C5 carbon of the diene fragment on the C2 position of the quinoleine framework through its diastereotopic (C1Si, C2Re) face, via **TS1nw**, has a different relative configuration than Mulzer’s experimental IMDA CA **2** (see **3** in Scheme 2 and **2** in Scheme 1 and Scheme 2) [5]. Note that CA **2** is formed via *exo* **TS2xw**. The computed entire *endo* and high facial selectivities, which are reproduced at all computational levels, including B3LYP/6-31G(d) and M05-2X/6-311+G(d,p) calculations (see Tables S2 and S4 in Supporting Information), makes it possible to assert that *endo* CA **3** is the diastereoisomeric product of the IMDA reaction of **1w**.

The geometries, including the lengths of the two forming bonds and relative energies of the TSs of the IMDA reactions of **1w** are shown in Figure 2. The asynchronicity at the TSs is: 0.36 for **TS1nw**, 0.18 for **TS1xw**, 0.39 at **TS2nw** and −0.02 at **TS2xw**. The *endo* TSs present a larger asynchronicity than the *exo* ones. Explicit solvation notably increases the asynchronicity at the most favorable *endo* TSs.

The CT at the TSs is: 0.30*e* at **TS1nw**, 0.27*e* at **TS1xw**, 0.31*e* at **TS2nw** and 0.29*e* at **TS2xw**. Therefore, the explicit inclusion of three water molecules increases the asynchronicity and the CT along a more polar process. This behavior provides an explanation of the catalytic role of the water HB formation, which accelerates the reaction through a more polar process [41].

**Figure 3:**
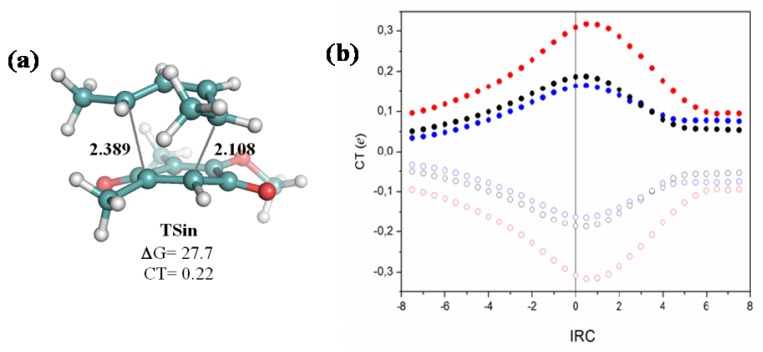
(**a**) Geometry of **TSin**. Bond distances in Å units. Relative energies ΔG in kcal/mol and CT in *e* units; (**b**) Graphical representation of the CT along the reaction coordinates. In blue IMDA reaction (**TS1n**), in black intermolecular DA (**TSin**) and red IMDA reactions with explicit solvent (**TS1nw**).

Recently, we have shown that the analysis of the change of the CT patterns along the reaction coordinate may be used to compare the electronic nature of the process in IMDA reactions [24,42] compared to their corresponding intermolecular processes. The intermolecular process has been studied using the TS associated with the more favourable *endo* channel for the DA reaction between (2*Z*,4*E*)-hexa-2,4-diene and 3,5-dimethy-2-methoxy-1,4-benzoquinone (**TSin** in Figure 3a). The CT analysis along the most favourable reactive channels is shown in Figure 3b.

The analysis of the CT change along the reaction coordinate indicates the similarity of the electronic process for both intra and intermolecular uncatalyzed processes. The catalyzed process shows a substantial increment in CT observed in the vicinity of the corresponding TS. Note that most polar DA reactions have a *two-stage one-step* mechanism through high asynchronous TSs [41]. Along the first stage of the reaction, the CT increases to reach the formation of the first C–C σ bond. At the second stage of the reaction, there is a decrease of the CT as a consequence of a back-donation along the formation of the second C–C σ bond [41]. This behaviour indicates that for these polar DA reactions the maximum CT at the most favourable regiosiomeric channels occurs after passing the TSs, while for the non-favoured TSs it appears at the TS region. Similar results have previously been reported for DA reactions of quinones catalyzed by Lewis acids [43].

The net result is the change in the reaction mechanism from non-polar to polar process. Therefore, explicit plus implicit solvent effects allow for an explanation of the acceleration observed in these polar DA reactions [41].

*NBO analysis of the favoured **TS1nw** channel:* NBO analysis has been used for a better understanding of donor-acceptor interactions [44,45]. This analysis shows that the XH bond length in an XHO complex is controlled by a balance between two main factors acting in opposite directions: the hyperconjugative interaction from the lone pair of oxygen to the σ*(X-H) antibonding orbital, leading to an elongation of the X–H bond, and the increase of the s character and polarization of the XH bond, which could lead to a shortening of the X–H distance. Table 1 and Table 2 summarize the results of the NBO analysis for HO-donor molecules after complexation with the reactant and the TS. Table 1 reports the distances and atomic charges of the C=O···HO group for the reactant in interaction with three water molecules. Bond distances and atom numbering are also defined in Table 1.

HB distances for the complex formed between the TS and water molecules decrease in comparison with the complex formed at the reactant. The most important effect is produced in d1, because this distance presents a large decrease in either case. In addition, the electronic charge in the hydrogen atoms shows a decrease upon formation of the complex, but the OH polarization is increased. The total positive charge in the H4 atom correlates well with the shortening of the d1 distances and the lowering of the complexation energy in both complex models. In addition, the MeO···HO interaction distance (d4 in Table 1) becomes significant at **TS1nw**.

Table 2 shows the occupation of the σ* antibonding orbitals of the O–H bonds, the variation of the percentage of the s character of selected bonds upon complexation, and the second order energies E(2) of the hyperconjugative interactions n(O)→σ*(O–H) [44].

The percentage of s character in the O–H bond in the water molecule that promotes the C=O···HO interaction is also depicted for the complexation process in the reactant and TS stages of the reaction. The decrease of electronic charge in H implies an increase in the percentage of the s character of the O–H bond, in agreement with Bent’s rule [46].

An important result of the NBO analysis is that the occupation of the σ*(O–H) orbital increases upon the complex formation with the reactant and TS structures due to the strength of the HB interaction. Hyperconjugative energies, n(O)→σ*(O–H), calculated by second-order perturbation theory E(2), show that the d1 interaction is responsible for the catalytic effect. This energy is moderate, 6.7 kcal/mol for the complex at the reactant, but significantly increases at the TS: 11.9 kcal/mol. Note that d2 does not show significant changes. However, the increase in interaction energy from 4.9 to 5.8 kcal/mol indicates that this interaction is favoured. However, the d3 and d4 interaction distances are interesting to analyze: the associated energies changes from reactants to TS are 4.0 kcal/mol to 3.8 kcal/mol in d3 and 0.2 kcal/mol to 4.9 kcal/mol in d4, respectively. These values indicate that the MeO···HO interaction becomes important at the TSs and therefore both interactions, C=O···HO for H4 and MeO···HO for H7, may become determinant in the explanation of the observed acceleration in these IMDA processes. These results show that the stabilization of the TS promoted by the presence of water molecules together with the polarity of the solvent are responsible for the catalytic activity experimentally observed in these systems [45].

**Figure 4:**
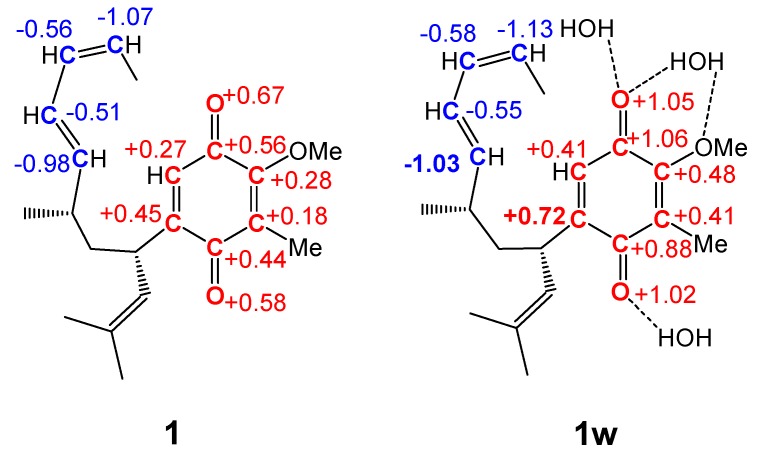
RMMRs of compound **1** and **1w**. R_k_ > 0, in red, correspond to electrophilic centres, while R_k_ < 0, in blue, and correspond to nucleophilic centres.

*Analysis based on DFT reactivity indices:* recent studies carried out on IMDA reactions [23,24] have shown that the reactivity indices defined within the conceptual DFT are powerful tools to study the polar character of these intramolecular processes. In Table 3 we report the static global, local and group properties, namely electronic chemical potential μ, chemical hardness η, global electrophilicity ω, global nucleophilicity *N*, of **1** and **1w**, while the local reactivity difference indices R*_k_* are given in Figure 4.

The global electrophilicity ω of quinone **1** is 3.65 eV, a value that falls within the range of strong electrophiles according to the electrophilicity scale [31]. Coordination by three water molecules at both the carbonyl and methoxy oxygen atoms notably increases the global electrophilicity of **1w** to 6.39 eV. On the other hand, quinones **1** and **1w** also present high nucleophilicity indices, *N =* 3.46 and 3.64 eV, respectively, also being classified as strong nucleophiles according the nucleophilicity scale [47]. Interestingly, explicit solvent effects increase the global electrophilicity of **1** notably, and slightly increase its nucleophilicity index. Note that in simple molecules involved in intermolecular processes, the increase of electrophilicity is accompanied by a decrease of the nucleophilicity [31].

Along a polar reaction involving asymmetric reagents, the most favourable reactive channel is that involving the two-centre interaction between the most electrophilic and nucleophilic centre of both reagents [41]. Recently, we have proposed a local reactivity difference index Rk to be able to predict the local electrophilic and/or nucleophilic activation within an organic molecule [34]. Together with the electrophilic and/or nucleophilic behaviour of the k centre, characterized by its sign, the magnitude of the Rk index accounts for the extent of the electronic activation. The representation of the more significant Rk indices, |Rk| > 0.10 eV, in a molecule constitutes the Rk molecular map of reactivity (RMMR) [34]. Studies devoted to intramolecular Michael reactions have shown that Rk indices are powerful tools in the understanding of intramolecular polar processes [48]. The RMMRs of quinone **1** and the water complex **1w** are given in Figure 4.

Analysis of the RMMRs of **1** and **1w** indicates that they present two separate electrophilic (atoms in red) and nucleophilic (atoms in blue) frameworks. While the electrophilic framework is associated with the quinone substructure, the nucleophilic framework is associated with the butadiene framework present in the chain. Note that the isobutene framework has no nucleophilic activation. A comparison of the R_k_ values of **1** and **1w** indicates that while explicit solvent effects slightly increase the nucleophilic R_k_ values, below 0.1 eV, they increase the electrophilic R_k_ values approximately twice, in clear agreement with the catalytic effect of the water molecules, illustrated by the increase of the global electrophilicity index.

The most electrophilic centres of **1** and **1w** are the two quinone oxygen atoms, while the most nucleophilic centres are the terminal carbon atoms of the butadiene framework. Consequently, the most favourable reactive channel should be that associated with the bond formation between these oxygen and carbon atoms [41,48]. However, the intramolecular nature of the process prevents the corresponding approach modes due to the strain developed along the intramolecular reaction. Taking into account the dienophilic framework of the quinone system present in **1** and **1w**, the C1–C2 double bond is more electrophilically activated than the C3–C4 one. In addition, in both molecules the C1 is the most electrophilically activated of the four carbon atoms. Consequently, the C1 carbon is the most electrophilic centres involved in these IMDA reactions. This behaviour is in complete agreement with the asynchronicity found in the C–C bond formation at the most favourable TSs.

These behaviours could be understood considering that the electrophilic activation promoted by the HB formation through water molecules only affects to the electrophilic quinone framework of **1**. A probable mechanism for this activation could be traced to the electron pull effect of water molecules towards the carbonyl oxygen atom which can be facilitated by the presence of the conjugated π system helping the creation of an electrophilic hole at the quinone framework of **1**.

Compounds **1** and **1w** both present the electrophilic patterns concentrated at the Dp fragments, ω_D_ = 3.64 and 6.26 eV, while the nucleophilic patterns are concentrated at the D fragments, *Ν*_D_ = 3.44 and 3.29 eV, respectively, in clear agreement with the RMMRs of **1** and **1w** given in Figure 4.

The transferability index ω_F_/ω accounts for the degree of projection of the global property onto the fragment. Compounds **1** and **1w** show a high degree of transferability of the fragment’s electrophilicity (ω_Dp_/ω) at the Dp fragment, 99.7 and 97.6 per cent, respectively. These results may be traced to the strong electrophilic character of the quinone system. In addition, these compounds present the nucleophilicity pattern concentrated at the D fragment. The degree of transferability of the fragment’s nucleophilicity (N_D_/N) is 96.0 at **1** and 99.4 at **1w**. Finally, the invariance of the electrophilicity index ω_Ω_ [24] accounts for the transferability of the fragment’s electrophilicity indices in comparison with the isolated 3,5-dimethy-2-methoxy-1,4-benzoquinone 7 (ω = 3.40 eV). The values of the ω_Ω_ index for the D fragments in compounds **1** and **1w** are 0.81 and 0.94 eV, respectively, which are very close to the electrophilicity values of isolated (2*Z*,4*E*)-hexa-2,4-diene 6 (ω = 0.82 eV). The reference values for the isolated quinones associated with **1** and **1w** are 3.10 eV and 6.34 eV, respectively. These values also are very close to the electrophilicity values of the quinone fragments in the IMDA reagents **1** and **1w**. Note that the high increment of the ω_Ω_ value of the Dp fragment in **1w** is due to the HB formation by water.

For intermolecular DA reactions, analysis of the electronic chemical potential μ of the reagents allows for the characterization of the electronic flux along a polar reaction, which takes place from the reagent with higher μ to the reagent with lower μ; but for an IMDA this analysis is not feasible. Recently, we have proposed that for intramolecular reactions, the analysis of the Ei indices can be used to characterize the direction of the electronic flux in an intramolecular process; that is, if the CT takes place from the D to the Dp fragment, DDpF, or from the Dp to the D fragment, DpDF [23]. The E1 and E2 indices are compiled in Table 3. For compounds **1** and **1w**, the corresponding values are: E1 = 7.08 and 9.55 eV, respectively. Note that the E2 values are zero in the two compounds. Therefore, for these compounds E1 > E2 thereby indicating that in these IMDA reactions the CT will take place from the D to the Dp fragment: these reactions present a DDpF electronic flux, in clear agreement with the CT found at the TSs [23].

For intermolecular DA reactions involving single molecules, the polar character of the reaction can be related with the Δω of reagents [24]. In the cases of IMDA reactions, the fragment’s electrophilicity difference Δω_Ω_ = |ω_Ω__B_ − ω_Ω__A_| can be used to estimate the polar character of the reaction. For compound **1** and **1w** these values are 2.39 eV and 5.40 eV, respectively. Therefore, the high Δω_Ω_ value found at complex **1w** indicates that the IMDA reaction of quinone **1** in water will have a large polar character induced by HB formation with water molecules, and consequently, this IMDA process will take place via a polar mechanism with substantial CT at the TS, and with a significant reduction in activation energy [41].

## 4. Concluding Remarks

The mechanism of the IMDA reaction of benzoquinone **1**, in the absence and in the presence of three water molecules has been studied at the M05-2X/6-31G(d) and B3LYP/6-31G(d) level of theory. The results obtained by the full exploration of the PES are complemented with a population analysis using the NBO method and a reactivity indices analysis based on group electrophilicity and nucleophilicity. Three water molecules have been incorporated to describe the catalysis by solvent molecules. HB donors act by stabilizing charges at the TSs, thus promoting a highly polar mechanism for these IMDA reactions. Both implicit and explicit solvation provokes the reduction of activation energies at the TSs of IMDA processes through the increase of the polarity of the reaction. These results are reinforced by the analysis of the reactivity indices showing that high electrophilicity/nucleophilicity fragment differences also explain highly polar processes. The intramolecular electrophilicity and nucleophilicity patterns show a remarkable transferability from their intermolecular counterparts. Large polarization at the TSs promoted by hydrogen-bonds and implicit solvation by water together with a high electrophilicity-nucleophilicity difference consistently explains the catalytic effects of water molecules.

## Figures and Tables

**Table 1:**
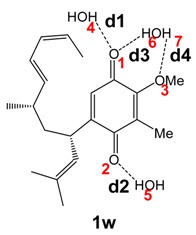
Distance of water molecule interactions (Å) and NBO atomic charges (e) in the reactant (R) the TS.

	Distance			Charge		
	1w	TS1nw		1	1w	TS1nw
d1	1.965	1.889	1	−0.533	−0.585	−0.673
d2	2.012	1.963	2	−0.545	−0.561	−0.610
d3	2.092	2.032	3	−0.538	−0.561	−0.581
d4	2.341	2.104	4		0.487	0.505
			5		0.495	0.498
			6		0.487	0.491
			7		0.477	0.486

**Table 2:** NBO analysis of the s character in the O–H bond, occupation of the antibonding σ*(H–O) orbital and hyperconjugative energies (kcal/mol).

	% H–O s character	σ*(H–O) Occupation	E(2)	% H–O s character	σ*(H–O) Occupation	E(2)
		1w			TS1nw	
d1	24.2	0.0218	6.7	23.3	0.0334	11.9
d2	24.6	0.0159	4.9	24.3	0.0201	5.8
d3	25.0	0.0140	4.0	24.7	0.0162	3.8
d4	25.9	0.0037	0.2	25.2	0.0109	4.9

**Table 3:**
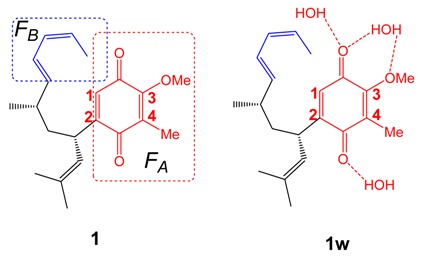
Global and regional indices. Electronic chemical potential *μ* and hardness *η*, in au, and electrophilicity *ω* and nucleophilicity *Ν*, in eV units.

	*μ*	*η*	*ω*	*N*	*ω_Dp_*	*ω_D_*	*N_Dp_*	*N_D_*	*E1*	*E2*
**1**	−0.1607	0.0940	3.65	3.46	3.64	0.00	0.00	3.44	7.08	0.00
**1w**	−0.1705	0.0619	6.39	3.64	6.26	0.00	0.00	3.29	9.55	0.00
	*F_A_*				*F_B_*					
	*μ_A_*	*η_A_*	*ω_A_*		*μ_B_*	*η_B_*	*ω_B_*		*Δω_Ω_*	
**1**	−0.1646	0.1189	3.10		−0.1064	0.1901	0.81		2.39	
**1w**	−0.1869	0.0749	6.34		−0.1091	0.1726	0.94		5.40	

## Supplementary Materials

Supplementary materials can be accessed at: http://www.mdpi.com/1420-3049/17/11/13687/s1.
