# Supplementary material for: On the Catalytic Effect of Water in the Intramolecular Diels–Alder Reaction of Quinone Systems: A Theoretical Study
Source: Molecules. 2012 Nov 20;17(11):13687–703. doi: 10.3390/molecules171113687 (PMC6268198; doi:10.3390/molecules171113687)
Supplement: Supplementary file 1 [file molecules-17-13687-s001.pdf]

Supporting Information

S1 **Table S1.** M05-2X/6-31(d) and B3LYP/6-31(d) total and relative energies of the stationary points involved in the IMDA reactions of **1** and **1w**.

S2 **Table S2**. M05-2X/6-31(d) and B3LYP/6-31(d) total and relative free energies for the stationary points involved in the IMDA reaction of **1**.

S2 **Table S3**. M05-2X/6-32G(d) total and relative free energies of the stationary points involved in the IMDA reactions of **1** and **1w**.

S4 **Table S4**. M05-2X/6-311+G(d,p)//M05-2X/6-32G(d) total and relative activation free energies in water of the stationary points involved in the IMDA reaction **1w**.

S4 M05-2X/6-31G(d) computed total energies, unique frequency imaginary, and cartesian coordinates of the stationary points involved uncatalyzed and water catalyzed reactions of **1**.

Table S1. Total (E, in au) and relative [a] (ΔE, in kcal/mol) energies of the stationary points involved in the IMDA reaction of **1** and **1w**.

	**M05-2X/6-31(d)**		**B3LYP/6-31(d)**	
	**E**	**ΔE**	**E**	**ΔE**
**1**	−1042.543999		−1042.686309	
**TS1n**	−1042.517296	16.76	−1042.642032	27.8
**TS1x**	−1042.506665	23.43	−1042.635465	31.9
**TS2n**	−1042.513860	18.91	−1042.641058	28.4
**TS2x**	−1042.509004	21.96	−1042.639008	29.7
**3**	−1042.597937	−33.85	−1042.716055	−18.7
**4**	−1042.607228	−39.68	−1042.716131	−18.7
**5**	−1042.599086	−34.57	−1042.713202	−16.9
**2**	−1042.608707	−40.60	−1042.713229	−16.9
**1w**	−1271.766477		−1271.947033	
**TS1nw**	−1271.752046	9.06	−1271.915943	19.5
**TS1xw**	−1271.739294	17.06	−1271.905249	26.2
**TS2nw**	−1271.746151	12.75	−1271.913825	20.8
**TS2xw**	−1271.743739	14.27	−1271.907296	24.9
**3w**	−1271.831450	−40.77	−1271.980356	−20.9
**4w**	−1271.839078	−45.56	−1271.986505	−24.8
**5w**	−1271.830886	−40.42	−1271.976899	−18.7
**2w**	−1271.839265	−45.68	−1271.988222	−25.8

[a] Relative to **1** or **1w**.

Table S2. Total (G, in au) and relative (ΔG, in kcal/mol, relative to **1**) free energies of the stationary points involved in the IMDA reaction of **1**.

	**M05-2X/6-31G(d)**		**B3LYP/6-31(d)**		**M05-2X/6-31G(d)/B3LYP /6-31(d)**	
	**G**	**ΔG**	**G**	**ΔG**	**G**	**ΔG**
**1**	−1042.152587		−1042.305227		−1042.146712	
**TS1n**	−1042.116588	22.6	−1042.252174	33.3	−1042.110583	22.7
**TS1x**	−1042.106244	29.1	−1042.245400	37.5	−1042.102081	28.0
**TS2n**	−1042.115550	23.2	−1042.250393	34.4	−1042.107928	24.3
**TS2x**	−1042.108741	27.5	−1042.249661	34.9	−1042.105609	25.8
**3**	−1042.192378	−25.0	−1042.312786	−4.7	−1042.189695	−27.0
**4**	−1042.200722	−30.2	−1042.324497	−12.1	−1042.200091	−33.5
**5**	−1042.193518	−25.7	−1042.315931	−6.7	−1042.190981	−27.8
**2**	−1042.203653	−32.0	−1042.327647	−14.1	−1042.200231	−33.6

Table S3. M05-2X/6-32G(d) total (G, in au) and relative [a] (ΔG, in kcal/mol) free energies of the stationary points involved in the IMDA reactions of **1** and **1w**.

	**gas phase**		**in water**	
	G	ΔG	G	ΔG
**1**	−1042.152587		−1042.160721	
**TS1n**	−1042.116588	22.6	−1042.128293	20.3
**TS1x**	−1042.106244	29.1	−1042.114524	29.0
**TS2n**	−1042.115550	23.2	−1042.124032	23.0
**TS2x**	−1042.108741	27.5	−1042.119031	26.2
**3**	−1042.192378	−25.0	−1042.203835	−27.1
**4**	−1042.200722	−30.2	−1042.211380	−31.8
**5**	−1042.193518	−25.7	−1042.203543	−26.9
**2**	−1042.203653	−32.0	−1042.211384	−31.8
**1w**	−1271.312651		−1271.336051	0.0
**TS1nw**	−1271.290386	14.0	−1271.307670	17.8
**TS1xw**	−1271.276130	22.9	−1271.294950	25.8
**TS2nw**	−1271.280983	19.9	−1271.303063	20.7
**TS2xw**	−1271.279108	21.0	−1271.300125	22.5
**3w**	−1271.362218	−31.1	−1271.385108	−30.8
**4w**	−1271.367543	−34.4	−1271.389476	−33.5
**5w**	−1271.359593	−29.5	−1271.380442	−27.9
**2w**	−1271.369113	−35.4	−1271.388153	−32.7

[a] Relative to **1** or **1w**.

Table S4. M05-2X/6-311+G(d,p)//M05-2X/6-32G(d) total (G, in au) and relative (ΔG, in kcal/mol, relative to **1**) activation free energies in water of the stationary points involved in the IMDA reaction **1w**.

	G	ΔG
**1w**	−1271.753130	
**TS1nw**	−1271.724829	17.8
**TS1xw**	−1271.713641	24.8
**TS2nw**	−1271.721860	19.6
**TS2xw**	−1271.715968	23.3
**3w**	−1271.796581	−27.3
**4w**	−1271.802322	−30.9
**5w**	−1271.790835	−23.7
**2w**	−1271.804916	−32.5

M05-2X/6-31G(d) computed total energies, unique frequency imaginary, and cartesian coordinates of the stationary points involved uncatalyzed and water catalyzed reactions of **1**.

1
E(RM052X) = −1042.543999 a.u.

C	0.00528700	−0.01499400	−0.01099700
H	0.00190400	−0.01257800	1.08329800
C	1.50362800	−0.01524900	−0.41484900
H	1.88457300	1.00286300	−0.27886600
H	2.03931800	−0.65616400	0.28674200
C	−0.72564700	1.21143900	−0.50950900
H	−0.24779700	1.76593600	−1.30978900
C	−1.89568100	1.66028200	−0.04602400
C	1.83744700	−0.48999800	−1.83903500
H	1.44819900	−1.50454400	−1.97469800
C	3.36321400	−0.53400400	−2.00726500
H	3.81813100	−1.17906100	−1.25244200
H	3.63150200	−0.90883500	−2.99603100
H	3.78824100	0.46734000	−1.89688900
C	1.24343300	0.38760500	−2.90444700
H	1.52766200	1.43802600	−2.86560500
C	0.42239000	−0.03039800	−3.87420200
H	0.10168900	−1.07024500	−3.86654600
C	−0.14696400	0.81233000	−4.93506800
H	−1.17802800	0.59324400	−5.19783600
C	0.47454900	1.78193800	−5.61823900
H	−0.10852700	2.31580600	−6.36247800
C	−0.64814600	−1.31278500	−0.42620200
C	−1.56870200	−1.43066500	−1.38929400
H	−1.93624800	−0.57399100	−1.93960000
C	−2.09012000	−2.74028200	−1.82631500
C	−0.21009000	−2.54684800	0.30825000
C	−0.75238500	−3.86948600	−0.07626600
C	−1.66751600	−3.96043100	−1.06353200
O	−2.81612600	−2.82686300	−2.80051100
O	0.57479600	−2.45440400	1.23855900
C	−2.68967800	0.97174600	1.03168500

H	−2.12908300	0.18476300	1.53417200
H	−3.02024100	1.69460400	1.78246400
H	−3.59067100	0.52049200	0.60396700
C	−2.53296900	2.90316100	−0.60817800
H	−2.66119100	3.65811600	0.17313700
H	−1.93352700	3.33296900	−1.41074000
H	−3.52958400	2.68176000	−1.00179000
C	1.91356200	2.19151300	−5.49867200
H	2.33052400	2.39740200	−6.48662000
H	2.02099200	3.10777900	−4.90970400
H	2.50881600	1.41380700	−5.01979800
C	−0.24166100	−5.05057300	0.69021800
H	−0.31738700	−4.86012800	1.76118300
H	−0.79961300	−5.94576900	0.42947500
H	0.81601200	−5.21393800	0.47224700
O	−2.11524300	−5.16911000	−1.45101500
C	−3.47218300	−5.33364900	−1.88970600
H	−3.72668900	−6.36209400	−1.64386300
H	−4.13757000	−4.65331000	−1.35947100
H	−3.55289200	−5.16283700	−2.95833600

1w
E(RM052X) = −1271.766477 a.u.

C	0.02307200	0.01056700	−0.00244400
H	0.01914500	0.02388900	1.09156000
C	1.52488400	−0.03224700	−0.39491300
H	1.94888000	0.95237900	−0.17036500
H	2.02061000	−0.74816700	0.26450500
C	−0.69126300	1.24476000	−0.50784200
H	−0.18525200	1.82175700	−1.27183800
C	−1.88519100	1.66928100	−0.08436500
C	1.86477700	−0.40226700	−1.85011700
H	1.38054800	−1.35185900	−2.09999100
C	3.38379700	−0.58367000	−1.97729300
H	3.74524800	−1.35299300	−1.29140500
H	3.65340300	−0.86885300	−2.99509400
H	3.89997200	0.35035200	−1.73952600
C	1.39578200	0.63926700	−2.82653000
H	1.82283300	1.63292600	−2.69604100
C	0.49323600	0.44207400	−3.79233300
H	0.01593400	−0.53149700	−3.87053100
C	−0.00623700	1.47428000	−4.71253200
H	−1.07964400	1.45230500	−4.87313400
C	0.71869700	2.39885700	−5.35416600
H	0.18120400	3.10302300	−5.98221800
C	−0.65068600	−1.28072600	−0.40168900
C	−1.49790800	−1.41555200	−1.43192100
H	−1.78119400	−0.57753300	−2.05864100
C	−2.03757800	−2.73109500	−1.81142700
C	−0.32585200	−2.48520600	0.43510600
C	−0.87192900	−3.82250700	0.08009400
C	−1.71094500	−3.91915800	−0.96555300
O	−2.72223900	−2.87307300	−2.81867100
O	0.37213600	−2.35649800	1.42951400

C	−2.71440800	0.95210400	0.94746500
H	−3.59191300	0.49676300	0.47742200
H	−2.16453100	0.16548600	1.46374200
H	−3.08477600	1.65933800	1.69434100
C	−2.51325300	2.91452100	−0.65053400
H	−2.68534600	3.65334200	0.13776600
H	−1.88535200	3.36393300	−1.41962900
H	−3.48729200	2.68594700	−1.09272900
C	2.21180100	2.55230300	−5.33037000
H	2.69782100	1.65216300	−4.95430000
H	2.58698200	2.76281900	−6.33420700
H	2.51594400	3.38995900	−4.69502600
C	−0.46641100	−4.98197100	0.93296300
H	−0.74803300	−4.81540000	1.97439700
H	−0.92136100	−5.89720900	0.56323800
H	0.62035000	−5.09163300	0.91952000
O	−2.21531000	−5.10717800	−1.37792500
C	−3.62786300	−5.27514700	−1.14412000
H	−3.92424800	−6.14577300	−1.71846000
H	−3.79984400	−5.42749700	−0.07784600
H	−4.18069300	−4.40725500	−1.49996800
H	−2.88831900	−1.30767900	−3.99623900
O	−2.66478300	−0.47364000	−4.43616800
H	−2.26099100	−0.73575000	−5.27061300
H	−0.20988300	−3.19058400	4.48490400
O	0.19115900	−3.87034000	3.93355100
H	0.49807800	−3.39525400	3.14863500
H	−3.07539000	−4.66859900	−3.83425200
O	−3.01423800	−5.59268900	−4.11190800
H	−2.28412300	−5.91825900	−3.57338500

2

E(RM052X) = −1042.608707 a.u.

C	0.05381300	−0.01011900	−0.01020600
H	0.13970300	0.00178000	1.07895800
C	1.47669300	−0.03811100	−0.57005400
H	1.45508900	0.00289000	−1.66299200
H	2.07749900	0.79663500	−0.20398200
C	−0.77946300	1.15024000	−0.46163200
H	−0.74962400	1.34828400	−1.53234300
C	−1.52471200	1.96334200	0.29720200
C	1.99187900	−1.40076700	−0.10667400
H	2.20137700	−1.33863500	0.96814000
C	3.24061500	−1.88074500	−0.83528900
H	4.07938800	−1.20131700	−0.67036900
H	3.54257000	−2.87479800	−0.49610600
H	3.04729200	−1.93365500	−1.90962200
C	0.77935700	−2.34458100	−0.31057500
H	0.89411500	−2.78986700	−1.30338700
C	0.72794800	−3.45981300	0.69431300
H	1.68261700	−3.90050500	0.96835700
C	−0.40020900	−3.94557000	1.20660000
H	−0.37545600	−4.78623900	1.89168600
C	−1.74481600	−3.37526800	0.83635600

H	−2.43826000	−3.50802800	1.67121700
C	−0.49967100	−1.44884900	−0.35641800
C	−1.57814400	−1.84382300	0.66059900
H	−1.27726800	−1.47608700	1.64422700
C	−2.93108700	−1.22609300	0.39996700
C	−1.03472700	−1.39729400	−1.78327600
C	−2.46782600	−1.09380400	−2.03754900
C	−3.33578900	−0.97842000	−1.01520400
O	−3.71163200	−1.01954800	1.30978100
O	−0.28841800	−1.51694900	−2.74096300
C	−1.71498900	1.84134700	1.78612300
H	−1.50336700	2.79814400	2.27164200
H	−2.75554300	1.58843200	2.00998200
H	−1.08790200	1.07930800	2.24407500
C	−2.26911600	3.11942600	−0.31952700
H	−2.17567300	3.12815500	−1.40608900
H	−3.33123000	3.07944100	−0.05993600
H	−1.88700000	4.06889800	0.06723900
C	−2.32005600	−4.10504200	−0.38504900
H	−2.35402200	−5.17769000	−0.18593100
H	−3.33222100	−3.77242800	−0.62195400
H	−1.68766300	−3.95668500	−1.26401900
C	−2.85678000	−0.90228700	−3.47021800
H	−2.28490400	−0.08003200	−3.90537600
H	−2.60134500	−1.79466200	−4.04391300
H	−3.92132400	−0.70193000	−3.55632500
O	−4.64885600	−0.71266000	−1.23437900
C	−5.06930200	0.59636000	−0.83046600
H	−6.14187800	0.63096900	−1.00226100
H	−4.85572700	0.76046000	0.22370100
H	−4.57122500	1.34987700	−1.44528700

3

E(RM052X) = −1042.597937 a.u.

C	0.04085700	−0.01566400	0.10534000
H	−0.13484900	0.06407000	1.17784600
C	1.57146000	−0.13944300	−0.17787800
H	1.86744800	0.64682100	−0.87940200
H	2.15908700	0.00651400	0.72905800
C	−0.58290700	1.16045800	−0.58955700
H	−0.29196600	1.28195400	−1.63212900
C	−1.48709900	2.01682600	−0.10166800
C	1.83430900	−1.52025900	−0.82269200
H	2.02965000	−2.26182100	−0.04976700
C	2.98647600	−1.50059400	−1.81979900
H	3.92006900	−1.20969700	−1.33356500
H	3.13917100	−2.48332600	−2.27151200
H	2.78744400	−0.78488100	−2.62316400
C	0.47565400	−1.81512700	−1.46118500
H	0.38266700	−1.08882200	−2.28684200
C	0.14275800	−3.16912900	−2.01389800
H	0.94168200	−3.80212000	−2.38485300
C	−1.12475900	−3.57575800	−2.07407300
H	−1.36130700	−4.55199400	−2.48734000

C	−2.31772400	−2.77455800	−1.60105800
H	−2.77430600	−3.33126200	−0.77264400
C	−0.55199800	−1.37979400	−0.41965500
C	−1.93346200	−1.35631900	−1.08133000
H	−1.92502600	−0.66334300	−1.92459800
C	−2.99329400	−0.86790900	−0.12885400
C	−0.64095100	−2.30532100	0.78824700
C	−1.81437900	−2.10388100	1.69813700
C	−2.85374900	−1.33509600	1.29636400
O	−3.94350200	−0.19675600	−0.46759600
O	0.21260300	−3.12069400	1.07947300
C	−2.03304600	2.00068300	1.30202400
H	−1.46986700	1.35173400	1.97162800
H	−2.02422700	3.01111100	1.71949600
H	−3.07376100	1.66131600	1.28696300
C	−2.10364500	3.06844000	−0.98479100
H	−1.98205200	4.06576800	−0.55278000
H	−1.66605300	3.06502500	−1.98326600
H	−3.17798100	2.88248400	−1.07870100
C	−3.36683400	−2.65639300	−2.71295600
H	−2.93242000	−2.16516900	−3.58614700
H	−3.71946800	−3.64351600	−3.01767600
H	−4.22259900	−2.06874000	−2.37729100
C	−1.76967100	−2.85515600	3.00091600
H	−1.10450000	−3.70698100	2.87317000
H	−1.37251100	−2.25080900	3.82003300
H	−2.76074400	−3.21137200	3.27886900
O	−3.91205400	−0.97502200	2.04635500
C	−3.70122400	−0.58932700	3.40394500
H	−3.81710800	−1.43300700	4.08205200
H	−2.70893900	−0.15305800	3.52394100
H	−4.46048500	0.15841700	3.62157600

4

E(RM052X) = −1042.607228 a.u.

C	0.07432200	0.01705500	−0.03936200
H	0.16148600	0.02933200	1.04949900
C	1.49772400	0.00212200	−0.59838800
H	1.47657400	0.07122400	−1.69284800
H	2.09635000	0.83392500	−0.22105600
C	−0.72740600	1.19431700	−0.50955500
H	−0.59197400	1.46192800	−1.55548500
C	−1.52801500	1.97839700	0.22375400
C	2.01923800	−1.36406600	−0.17028800
H	2.15929500	−1.33395500	0.91511800
C	3.34732600	−1.74329700	−0.81416600
H	4.11704200	−1.02254400	−0.53058800
H	3.68474700	−2.73199500	−0.49517700
H	3.27031700	−1.74309900	−1.90351200
C	0.81509600	−2.31738500	−0.44495900
H	0.73699300	−3.00823200	0.39769200
C	0.97498800	−3.15816300	−1.68302900
H	1.91490300	−3.69212800	−1.77104300
C	0.03603000	−3.35519900	−2.60417300

H	0.21541500	−4.04191800	−3.42511800
C	−1.31690000	−2.71026100	−2.52106400
H	−1.67312000	−2.47293500	−3.52740800
C	−0.46689400	−1.42067100	−0.43531900
C	−1.16916300	−1.34932700	−1.79763500
H	−0.57065400	−0.72984700	−2.46957100
C	−2.53770800	−0.71022700	−1.75453000
C	−1.41543500	−1.82405100	0.68229900
C	−2.84433300	−1.42554500	0.61039300
C	−3.34836400	−0.86377100	−0.50700000
O	−3.02059400	−0.20750600	−2.75075300
O	−1.00604000	−2.36555800	1.69565200
C	−1.84543800	1.76101300	1.67894100
H	−2.92737500	1.66376200	1.81259000
H	−1.37969200	0.87018500	2.09668800
H	−1.53078700	2.62675500	2.26935000
C	−2.17266800	3.19787800	−0.38230200
H	−1.78177600	4.10571100	0.08744600
H	−1.98841600	3.25831400	−1.45506300
H	−3.25294700	3.19696000	−0.21462000
C	−2.31997900	−3.68337000	−1.88332700
H	−2.32414600	−4.61836600	−2.44689800
H	−2.03482300	−3.92245800	−0.85606300
H	−3.33599000	−3.28419600	−1.88383100
C	−3.66569100	−1.69211700	1.83398400
H	−3.20631100	−1.21956500	2.70390600
H	−4.68140000	−1.32921800	1.70229200
H	−3.68566800	−2.76455700	2.03781700
O	−4.66694000	−0.56011500	−0.59134000
C	−4.98983500	0.81837700	−0.81004700
H	−6.04455200	0.83908800	−1.07157700
H	−4.83115100	1.38116300	0.11312500
H	−4.39773000	1.23326800	−1.62098100

5
E(RM052X) = −1042.599086 a.u.

C	0.02159600	0.00295800	−0.04505400
H	0.07719800	0.03112400	1.04708300
C	1.45902900	−0.00571500	−0.60791900
H	1.42187000	0.15655900	−1.68836600
H	2.06149500	0.79383300	−0.17427800
C	−0.81421900	1.14540300	−0.53774800
H	−0.71264800	1.36653100	−1.59936700
C	−1.63637100	1.92107400	0.18018700
C	2.03451400	−1.41545200	−0.31695300
H	2.65282000	−1.35716800	0.58264500
C	2.91795000	−1.95260800	−1.43970400
H	3.72303200	−1.24296500	−1.64606500
H	3.38222100	−2.90021400	−1.15665000
H	2.33968500	−2.10050400	−2.34875800
C	0.77482100	−2.25028100	0.04629200
H	0.72559600	−2.22129000	1.14295800
C	0.60316400	−3.69087500	−0.34088600
H	1.45493400	−4.27861700	−0.66211500

C	−0.61125400	−4.23459100	−0.26016600
H	−0.75947000	−5.27821300	−0.52080400
C	−1.86117500	−3.50113000	0.18023800
H	−2.59888800	−3.63470500	−0.61992100
C	−0.45837600	−1.44201700	−0.38786500
C	−1.65874100	−1.96174500	0.39982500
H	−1.49723300	−1.77218500	1.46476200
C	−2.94560300	−1.27645500	0.01741000
C	−0.76186000	−1.52331200	−1.87788800
C	−2.15064900	−1.22700200	−2.33747000
C	−3.14920900	−1.05510300	−1.44954500
O	−3.81566500	−1.00685800	0.82208700
O	0.09717100	−1.72263600	−2.71705000
C	−1.93736300	1.74949800	1.64536000
H	−1.31311400	1.00251800	2.13160500
H	−1.81126900	2.69999800	2.17113400
H	−2.97858200	1.43977000	1.77502700
C	−2.36024900	3.07604900	−0.46240000
H	−3.43751800	3.00924800	−0.28388600
H	−2.02746400	4.02245200	−0.02552200
H	−2.18600800	3.11531700	−1.53831000
C	−2.44986600	−4.12594800	1.45088900
H	−3.35167000	−3.59027800	1.75389100
H	−2.70499200	−5.17437900	1.28680100
H	−1.72431200	−4.07440400	2.26547600
C	−2.33791800	−1.08854400	−3.81626300
H	−2.01768800	−2.00474100	−4.31478500
H	−3.37843700	−0.88390700	−4.05357000
H	−1.70266600	−0.28794300	−4.20113200
O	−4.41253900	−0.76510900	−1.85381100
C	−4.84489500	0.56377900	−1.53595000
H	−5.87243500	0.63339600	−1.88318000
H	−4.80047300	0.73503600	−0.46242500
H	−4.22402600	1.29067800	−2.06498900

2w

E(RM052X) = −1271.839265 a.u.

C	−0.00309900	−0.03240100	−0.02506600
H	0.06608400	−0.01577600	1.06495500
C	1.42426900	−0.04649700	−0.57333600
H	1.40640200	0.01019000	−1.66623100
H	2.00984000	0.79493600	−0.19824800
C	−0.83053400	1.12358400	−0.50101500
H	−0.77018000	1.32105500	−1.57077200
C	−1.57016200	1.95059900	0.25012400
C	1.95597500	−1.40273600	−0.11166900
H	2.15860200	−1.33923400	0.96393400
C	3.21518100	−1.86429200	−0.83424500
H	4.04298300	−1.17289800	−0.66427900
H	3.53027700	−2.85415500	−0.49505600
H	3.02899700	−1.91939100	−1.90997600
C	0.75885100	−2.36427500	−0.32232500
H	0.88348400	−2.80742800	−1.31486400
C	0.71803500	−3.48486900	0.67753500

H	1.67689600	−3.91961600	0.94566200
C	−0.40443700	−3.98362200	1.18911100
H	−0.37012000	−4.83108400	1.86539500
C	−1.75728500	−3.42855100	0.82601800
H	−2.44453800	−3.56973400	1.66620100
C	−0.53247800	−1.48834500	−0.36809700
C	−1.59563900	−1.89196800	0.65922100
H	−1.28484800	−1.53217100	1.64177600
C	−2.94080100	−1.27601300	0.40599200
C	−1.07433800	−1.44196300	−1.78382200
C	−2.51020800	−1.13579700	−2.03257500
C	−3.36041200	−1.02664600	−0.99590000
O	−3.72392300	−1.04701700	1.32149400
O	−0.33024400	−1.56196900	−2.74876800
C	−1.78418900	1.84084400	1.73655400
H	−1.47094100	2.76622600	2.22913700
H	−2.85137000	1.71903200	1.95036500
H	−1.26700700	1.00888300	2.20895500
C	−2.25933300	3.13237300	−0.38427000
H	−2.13888700	3.13552100	−1.46823400
H	−3.32749000	3.13913200	−0.14639000
H	−1.84765300	4.06593200	0.01125900
C	−2.33990000	−4.16187300	−0.38888900
H	−2.32062200	−5.23725300	−0.20199300
H	−3.37520400	−3.87236200	−0.57275700
H	−1.73593500	−3.97976500	−1.28222700
C	−2.92022900	−0.91604000	−3.45397600
H	−2.44163600	−0.01556600	−3.84698700
H	−2.57940800	−1.75268500	−4.06566600
H	−4.00043800	−0.82143200	−3.52942500
O	−4.67246300	−0.70483400	−1.18624300
C	−4.96127200	0.67921200	−0.92782000
H	−6.02481000	0.80388900	−1.11192100
H	−4.72609600	0.92552400	0.10677100
H	−4.38402900	1.30646400	−1.60914900
H	0.57613800	1.29502000	−4.36511200
O	−0.25203500	1.00405600	−3.96941300
H	−0.14954200	0.05217400	−3.82086300
H	−2.14355200	−1.70166900	4.16845500
O	−2.12823000	−0.84366000	3.73073400
H	−2.83165500	−0.89173600	3.06316300
O	−5.56135900	−3.06416800	0.22194500
H	−5.65628700	−2.38577800	−0.45962500
H	−5.28288700	−2.54611300	0.98722000

3w

E(RM052X) = −1271.831450 a.u.

C	0.03406700	0.01599300	−0.00593200
H	0.01521200	0.05786500	1.08333600
C	1.51575700	0.00215900	−0.48437800
H	1.60754500	0.63524300	−1.37359300
H	2.18621500	0.40985500	0.27296400
C	−0.77178700	1.15009800	−0.56444100
H	−0.75896000	1.23410900	−1.64974500

C	−1.54355200	2.01472800	0.10692800
C	1.86093200	−1.44741700	−0.87778000
H	2.07964300	−2.03686400	0.01284400
C	3.02119000	−1.54087700	−1.86039500
H	3.93439500	−1.12701500	−1.42796200
H	3.22460300	−2.57708200	−2.13947800
H	2.79621000	−0.98114100	−2.77292400
C	0.51926400	−1.88849400	−1.46379400
H	0.39035400	−1.25721700	−2.35911800
C	0.22937700	−3.30184600	−1.87484400
H	1.04679900	−3.96436400	−2.13714700
C	−1.03365600	−3.72125500	−1.94630100
H	−1.25217900	−4.73840800	−2.25717900
C	−2.24866000	−2.88072300	−1.61382200
H	−2.76533500	−3.38048900	−0.78515300
C	−0.52823400	−1.39077800	−0.46966200
C	−1.88818000	−1.42498200	−1.17139500
H	−1.86459300	−0.79010300	−2.05734100
C	−2.97318000	−0.90708700	−0.27712900
C	−0.63725500	−2.22900300	0.79008600
C	−1.84268500	−2.01772500	1.64628200
C	−2.88753200	−1.29752600	1.16831300
O	−3.93770300	−0.27416300	−0.68254900
O	0.24629200	−2.98211700	1.16886200
C	−1.71812800	2.06793500	1.60100200
H	−1.15612300	1.30332000	2.13605700
H	−1.40049300	3.04599500	1.97523300
H	−2.78113100	1.96714300	1.83823700
C	−2.37239900	3.03204200	−0.63188400
H	−2.09926200	4.04805900	−0.33097800
H	−2.26809500	2.93441000	−1.71177100
H	−3.42385700	2.89071100	−0.36391900
C	−3.21777100	−2.82910800	−2.80137500
H	−2.72887700	−2.36742800	−3.66179700
H	−3.53699400	−3.83346500	−3.08476900
H	−4.10503300	−2.24413600	−2.55024800
C	−1.81453900	−2.67882600	2.99473900
H	−1.39760300	−3.68117000	2.89005800
H	−1.17650700	−2.13283600	3.69508300
H	−2.81309600	−2.76156700	3.41476300
O	−3.99078200	−0.93497100	1.84045300
C	−3.90367800	−0.44550200	3.18819200
H	−4.27062000	−1.20057200	3.88042800
H	−2.87477300	−0.18228900	3.42929600
H	−4.53736600	0.43679900	3.21612900
H	−3.46079800	0.63623300	−2.37435000
O	−3.01677500	0.88820500	−3.20020200
H	−3.55396900	0.49605200	−3.89731300
H	0.52128400	−5.52479200	3.11562600
O	−0.09693100	−5.59052000	2.37994400
H	0.10987800	−4.81722900	1.83378600
H	−4.89127200	0.79229900	0.75646800
O	−5.17174000	1.59339700	1.22878600
H	−5.70732400	2.08194000	0.59515200

4w
E(RM052X) = −1271.839078 a.u.

C	−0.04518600	−0.05280600	−0.00074400
H	−0.07840600	−0.04157300	1.09147000
C	1.42781000	−0.06522800	−0.42118200
H	1.50688100	0.06657200	−1.50751800
H	1.99271800	0.73637400	0.05880800
C	−0.78810200	1.11848500	−0.56966700
H	−0.63099900	1.29735900	−1.63045000
C	−1.54026200	2.01255400	0.08903100
C	1.89368100	−1.46153700	−0.03255400
H	1.86792500	−1.52297300	1.06136000
C	3.29994600	−1.80798900	−0.50547700
H	4.03017600	−1.16402500	−0.01167100
H	3.55902900	−2.84442100	−0.27592300
H	3.40094500	−1.66030600	−1.58265900
C	0.74889200	−2.37263500	−0.55007900
H	0.62992900	−3.19805700	0.15590100
C	1.00017000	−2.99695400	−1.89802200
H	1.99266000	−3.40044300	−2.06405100
C	0.06006200	−3.18524000	−2.82052700
H	0.28614200	−3.73137200	−3.73019100
C	−1.34959900	−2.70142200	−2.62242800
H	−1.78964800	−2.44940800	−3.59151400
C	−0.54440600	−1.49612600	−0.48357700
C	−1.26979800	−1.37282100	−1.82829600
H	−0.71679200	−0.68542400	−2.47203800
C	−2.65706600	−0.80185100	−1.71957500
C	−1.45698100	−1.97131200	0.62253500
C	−2.86609500	−1.50004100	0.65445500
C	−3.40389600	−0.92741800	−0.44015400
O	−3.21841600	−0.32928400	−2.70301700
O	−1.03654300	−2.63266600	1.56384100
C	−1.84117400	1.98551300	1.56310500
H	−2.92281200	2.02284200	1.72902900
H	−1.44859900	1.11542100	2.08553500
H	−1.43028700	2.88335700	2.03664000
C	−2.10767600	3.19574100	−0.65469100
H	−1.63827700	4.12114300	−0.30603400
H	−1.94469400	3.10592900	−1.72958800
H	−3.18010600	3.30207200	−0.46495800
C	−2.21873100	−3.78650700	−1.97162400
H	−2.14686700	−4.70538700	−2.55677500
H	−1.86347900	−4.01868500	−0.96410000
H	−3.27044500	−3.49544000	−1.93660700
C	−3.61871600	−1.71353500	1.92947000
H	−3.11520000	−1.19243800	2.74711700
H	−4.64133200	−1.35840300	1.83672100
H	−3.62020500	−2.77623200	2.17824200
O	−4.71142700	−0.54579300	−0.45428200
C	−4.94238700	0.86684700	−0.58800800
H	−6.01332300	0.97918800	−0.73377200
H	−4.63628900	1.37098700	0.32903200
H	−4.40176200	1.26532000	−1.44283500
H	−0.80886400	−1.52287100	3.12055900

O	−0.79267400	−0.65290600	3.54692800
H	−0.07308500	−0.68764300	4.18575800
H	−0.99838700	1.21112600	−4.79626200
O	−1.23213400	1.35749100	−3.87364600
H	−2.00200100	0.79046600	−3.70721200
O	−5.34460500	−2.37407700	−2.60858700
H	−5.53665000	−1.91409800	−1.78105100
H	−4.85248700	−1.70784300	−3.10463500

5w
E(RM052X) = −1271.830886 a.u.

C	−0.01311500	−0.01343400	−0.03558200
H	0.00486700	0.01124100	1.05778600
C	1.43904900	−0.01992200	−0.56098400
H	1.42433200	0.18274600	−1.63469600
H	2.03244700	0.76472700	−0.08897000
C	−0.82285500	1.13286100	−0.56576700
H	−0.66729800	1.35158900	−1.62159100
C	−1.66007300	1.91869600	0.12346000
C	2.00409500	−1.43849700	−0.29502200
H	2.61097700	−1.40366100	0.61317800
C	2.90306800	−1.95429000	−1.41567800
H	3.71132600	−1.24124500	−1.59731400
H	3.36328300	−2.90725300	−1.14498500
H	2.33952700	−2.08835900	−2.33621900
C	0.73895000	−2.27813100	0.03353300
H	0.67476100	−2.27427200	1.12915200
C	0.55907100	−3.70651900	−0.39687500
H	1.40252000	−4.28535000	−0.75414500
C	−0.65891500	−4.24390100	−0.31751500
H	−0.81890600	−5.27637400	−0.61267500
C	−1.89386000	−3.52455700	0.18197400
H	−2.68445600	−3.67150800	−0.56020200
C	−0.48848000	−1.45856900	−0.39617400
C	−1.68554100	−1.97990500	0.39550900
H	−1.51315100	−1.78608100	1.45620600
C	−2.96156400	−1.29994600	0.00191500
C	−0.78191100	−1.51515300	−1.88312100
C	−2.17244200	−1.24132700	−2.35347800
C	−3.16602800	−1.09296600	−1.45879200
O	−3.84375300	−0.99414200	0.79418200
O	0.09375300	−1.67626900	−2.72005800
C	−2.01394600	1.75857000	1.57896600
H	−1.60010000	0.86496800	2.04197800
H	−1.68437000	2.63496800	2.14591500
H	−3.10255600	1.70364800	1.68442800
C	−2.32381900	3.09546600	−0.54537400
H	−3.41193000	3.04769100	−0.43810400
H	−2.00493600	4.02970100	−0.07271500
H	−2.07715400	3.14703300	−1.60634600
C	−2.39797400	−4.14651300	1.49046300
H	−3.28847200	−3.62261100	1.84401500
H	−2.65252400	−5.19735400	1.34409500
H	−1.63647700	−4.07220300	2.26885000

C	−2.37402800	−1.09122100	−3.82843700
H	−1.91390600	−1.93399500	−4.34582100
H	−3.43412600	−1.04746700	−4.06496000
H	−1.87967100	−0.18451200	−4.18544200
O	−4.43980500	−0.79469700	−1.85158600
C	−4.78129100	0.58998700	−1.67700700
H	−5.79103400	0.70119700	−2.06285700
H	−4.74785300	0.85426200	−0.62127300
H	−4.08964300	1.21244600	−2.24737200
O	0.27732400	0.87940200	−3.96292000
H	0.37764900	−0.06980500	−3.79501700
H	1.16123400	1.19731100	−4.17540600
H	−2.87711900	−0.45273400	3.99499800
O	−2.61088800	−1.15518200	3.39228100
H	−3.20131300	−1.07109000	2.62545500
H	−5.28121800	−2.50115700	−1.13735800
O	−5.15523000	−3.29258100	−0.59504000
H	−5.20580700	−2.95161100	0.30533000

TS1n
E(RM052X) = −1042.517296 a.u.
1 imaginary frequency −376.255 cm^{-1}

C	0.13968200	0.01631200	0.05149100
H	0.16089900	0.13640200	1.13717000
C	1.61328500	−0.14525700	−0.42750200
H	1.95128600	0.81376200	−0.83066600
H	2.25174800	−0.38600800	0.41866200
C	−0.48667300	1.24201600	−0.57164600
H	−0.08811600	1.53312800	−1.54222000
C	−1.45169800	2.00748200	−0.05011600
C	1.76723700	−1.24541600	−1.50771100
H	2.15807200	−2.15253900	−1.04306200
C	2.72198000	−0.80680400	−2.62497600
H	3.69574500	−0.53975600	−2.20903400
H	2.86532900	−1.60319800	−3.35691600
H	2.32564600	0.06918200	−3.14548400
C	0.40006400	−1.52927200	−2.04871200
H	−0.12038000	−0.64287600	−2.38064600
C	−0.07124000	−2.75137600	−2.50350000
H	0.54385600	−3.63559600	−2.36552300
C	−1.43326600	−2.91785600	−2.79234500
H	−1.81795000	−3.93285900	−2.76806000
C	−2.35740400	−1.88142000	−2.79485000
H	−3.39881200	−2.18349000	−2.74330400
C	−0.67630200	−1.25964100	−0.21207300
C	−2.03685000	−1.25164300	−0.52477400
H	−2.54991500	−0.31276000	−0.68167300
C	−2.92159800	−2.36123200	−0.16561300
C	−0.15551600	−2.46742900	0.50335500
C	−0.94857300	−3.71194800	0.47418200
C	−2.25473900	−3.66732700	0.13364100
O	−4.13799400	−2.24446200	−0.10722400
O	0.91782500	−2.42990800	1.09155500
C	−2.11778900	1.74192000	1.27307000

H	−1.70896100	0.87469700	1.78780600
H	−2.02420100	2.61369600	1.92710000
H	−3.18820400	1.56893200	1.12484200
C	−1.96668800	3.21810100	−0.78376900
H	−1.83222700	4.12011400	−0.17952900
H	−1.45414000	3.36142700	−1.73542800
H	−3.03930000	3.12406000	−0.97779400
C	−2.15489400	−0.51934600	−3.43421400
H	−2.03554500	0.29934500	−2.72248100
H	−1.28830500	−0.52836300	−4.09673500
H	−3.03387000	−0.29193900	−4.04001400
C	−0.29994900	−4.98844500	0.92183900
H	0.75997800	−4.82043700	1.09659800
H	−0.75502100	−5.35005500	1.84647200
H	−0.43761000	−5.76831800	0.17053900
O	−2.96468200	−4.80775500	0.20690200
C	−4.20893200	−4.94790600	−0.48894000
H	−4.35449800	−6.02137300	−0.58606700
H	−5.02008000	−4.49541400	0.07193400
H	−4.15691300	−4.49251900	−1.47814300

TS1x
E(RM052X) = −1042.506665 a.u.

1 imaginary frequency −396.724 cm^{-1}

C	−0.02706600	−0.02897100	0.08303700
H	−0.11269600	0.04168200	1.16735100
C	1.49362600	−0.13105300	−0.25043100
H	1.74133100	0.64888600	−0.97848200
H	2.08812000	0.07899100	0.64043500
C	−0.60279300	1.20984900	−0.55002000
H	−0.43264800	1.29109200	−1.62261900
C	−1.26576100	2.20655500	0.04427300
C	1.89745100	−1.49438000	−0.83619300
H	1.98772100	−2.20552500	−0.01730600
C	3.24630800	−1.41614900	−1.56043300
H	4.02070700	−1.06267000	−0.87616600
H	3.54661100	−2.39383500	−1.94030100
H	3.19997100	−0.72397100	−2.40395600
C	0.76994800	−1.94523300	−1.72937400
H	0.45280200	−2.97555600	−1.65745500
C	0.39828000	−1.17365200	−2.81705900
H	0.99040200	−0.28204000	−3.00597700
C	−0.85044100	−1.21079200	−3.45343700
H	−1.10509200	−0.32489400	−4.02765900
C	−1.89738200	−2.06591900	−3.16295400
H	−2.87096200	−1.74614400	−3.52320700
C	−0.82336300	−1.29193100	−0.28681000
C	−2.05871000	−1.24175300	−0.92994000
H	−2.42806900	−0.30101300	−1.31375000
C	−3.11998900	−2.22669000	−0.68647400
C	−0.62379700	−2.41716100	0.67729600
C	−1.55273500	−3.57693200	0.67623100
C	−2.75264500	−3.46516200	0.07412100
O	−4.25545900	−2.07377800	−1.11375300

O	0.27890200	−2.38128700	1.50237000
C	−1.61164700	2.27029000	1.50818600
H	−2.69580500	2.35136200	1.63000700
H	−1.27404500	1.40304400	2.07099900
H	−1.17510800	3.16563700	1.96051400
C	−1.74083300	3.39585900	−0.75048200
H	−1.30567400	4.31989800	−0.35802700
H	−1.47358300	3.31015200	−1.80402000
H	−2.82733800	3.50082600	−0.67632100
C	−1.82157800	−3.55192600	−2.91869500
H	−1.54283900	−4.03734500	−3.85874800
H	−1.09870600	−3.85318700	−2.16683100
H	−2.79450700	−3.94157500	−2.61970000
C	−1.12147500	−4.78568300	1.44911900
H	−0.81502200	−4.48895500	2.45233800
H	−1.92652100	−5.51372900	1.50119600
H	−0.25221800	−5.25025700	0.97713300
O	−3.59756500	−4.51909800	0.04951700
C	−5.00069200	−4.31278700	0.28019200
H	−5.35326000	−5.23557500	0.73672800
H	−5.16223900	−3.47748600	0.95960900
H	−5.51990000	−4.12305100	−0.65366400

TS2n
E(RM052X) = −1042.513860 a.u.
1 imaginary frequency −390.018 cm^{-1}

C	−0.01223100	0.00794800	0.01739700
H	0.00241800	−0.02703400	1.10938800
C	1.44253000	−0.08018300	−0.49884200
H	1.46997900	0.27603700	−1.52634000
H	2.06657000	0.58765300	0.09800200
C	−0.66175900	1.29134800	−0.43293800
H	−0.63581700	1.44710600	−1.50739700
C	−1.25153800	2.21854000	0.32745600
C	1.98526100	−1.54066300	−0.45888800
H	2.77417100	−1.58469300	0.30119200
C	2.59062900	−1.98711600	−1.78848700
H	3.38076500	−1.29473800	−2.08488900
H	3.03084100	−2.98339300	−1.70557300
H	1.83917700	−1.99305700	−2.57777500
C	0.88020200	−2.42286000	0.05504800
H	0.61283100	−2.17649400	1.06979100
C	0.48122400	−3.67414500	−0.39315800
H	0.87279700	−4.06339600	−1.32650900
C	−0.65063400	−4.27696800	0.17284800
H	−1.15453400	−5.03362100	−0.42004100
C	−1.32560800	−3.76631200	1.27778400
H	−2.32396300	−4.16198200	1.43604600
C	−0.83477600	−1.20315000	−0.42751800
C	−1.88369400	−1.69149000	0.35815000
H	−2.07863500	−1.24243000	1.32557100
C	−3.05554700	−2.33943600	−0.23645900
C	−0.93160900	−1.44646600	−1.89860800
C	−1.86182300	−2.47787100	−2.39762400

C	−2.86799600	−2.91332400	−1.60890700
O	−4.12864900	−2.42935000	0.34061900
O	−0.24785600	−0.82049400	−2.70013000
C	−1.40215500	2.16452700	1.82514900
H	−2.46306600	2.15044100	2.09267600
H	−0.92944000	1.29691100	2.28150000
H	−0.97407000	3.06269100	2.27980900
C	−1.85551900	3.44856700	−0.30048100
H	−2.92410300	3.51201800	−0.07358100
H	−1.39128600	4.35476000	0.10044200
H	−1.73268100	3.44762000	−1.38335900
C	−0.66438400	−3.30074200	2.56593500
H	−0.75936800	−2.23262100	2.76731200
H	−1.14065300	−3.82064300	3.39922700
H	0.39418900	−3.56116500	2.57590900
C	−1.69335400	−2.89639500	−3.82669600
H	−0.65521100	−3.17744100	−4.01460100
H	−2.35043200	−3.72894600	−4.06418200
H	−1.91752800	−2.05993300	−4.49132300
O	−3.78258200	−3.76224000	−2.11362800
C	−4.51745200	−4.63460800	−1.24716000
H	−4.81469200	−5.47132000	−1.87522100
H	−3.88900700	−4.99588600	−0.43219700
H	−5.38602000	−4.13005400	−0.83710700

TS2x
E(RM052X) = −1042.509004 a.u.
1 imaginary frequency −402.932 cm^{-1}

C	0.06144000	0.02421500	0.01235900
H	0.05781800	0.01282200	1.10445200
C	1.51376000	−0.07068600	−0.47415100
H	1.53844900	0.09705000	−1.54894900
H	2.09889600	0.72286800	−0.00297600
C	−0.57512400	1.29396200	−0.49860600
H	−0.49934000	1.42443100	−1.57624100
C	−1.21426300	2.23592300	0.20084300
C	2.13616900	−1.46216400	−0.16674100
H	2.75309200	−1.36010200	0.73135300
C	3.01786400	−1.93530700	−1.32353600
H	3.80172400	−1.20316200	−1.52846800
H	3.49396700	−2.89118300	−1.09577300
H	2.41238500	−2.04561300	−2.22466500
C	1.04541700	−2.44904000	0.16836100
H	0.71520300	−3.11582400	−0.61468000
C	0.75723000	−2.69566900	1.50197300
H	1.38645800	−2.19222900	2.23089600
C	−0.43890200	−3.23194000	1.99085400
H	−0.65455400	−3.05376000	3.03944000
C	−1.48684300	−3.66794900	1.19485800
H	−2.43617600	−3.81159400	1.70068000
C	−0.79613200	−1.17403500	−0.40992800
C	−1.87007000	−1.56909500	0.38425100
H	−1.97650700	−1.14571400	1.37525200
C	−3.16873100	−1.97582100	−0.17879500

C	−0.90940500	−1.45280500	−1.87505400
C	−2.15818900	−2.03481100	−2.43502300
C	−3.21924200	−2.29169700	−1.64115700
O	−4.17706600	−2.02534600	0.50834000
O	0.00513300	−1.20061600	−2.65037000
C	−1.43555400	2.21885200	1.69037300
H	−1.05485800	3.14094500	2.13926900
H	−2.50724900	2.18012700	1.90810900
H	−0.95986400	1.37719500	2.18954300
C	−1.80371200	3.43822600	−0.49116200
H	−1.63338600	3.40675300	−1.56728900
H	−2.88191000	3.49478200	−0.31301000
H	−1.36825900	4.36215300	−0.09873600
C	−1.30632800	−4.48008000	−0.06732600
H	−0.47732500	−5.18011700	0.05894000
H	−2.20981500	−5.05344200	−0.27836200
H	−1.09863700	−3.88016000	−0.95173500
C	−2.15965500	−2.29356500	−3.91118400
H	−1.28308300	−2.88048000	−4.18833700
H	−3.06613700	−2.81394600	−4.20806700
H	−2.09028700	−1.35127400	−4.45832800
O	−4.36384900	−2.74458200	−2.18758200
C	−5.23729700	−3.60629200	−1.44632400
H	−5.76135600	−4.19185000	−2.19853800
H	−4.67049000	−4.26970700	−0.79323800
H	−5.93671900	−3.02723200	−0.85228000

TS1nw
E(RM052X) = −1271.752046 a.u.
1 imaginary frequency −344.881 cm^{-1}

C	−0.01903000	0.00967900	−0.01238000
H	−0.00202800	0.10666400	1.07598800
C	1.45426300	−0.06033100	−0.50925200
H	1.73168100	0.92089900	−0.90356100
H	2.12194100	−0.27425100	0.32214600
C	−0.73575600	1.19846200	−0.61047300
H	−0.35772300	1.54403600	−1.57089400
C	−1.78548800	1.84459800	−0.09108700
C	1.64571500	−1.13514800	−1.60839300
H	2.09158600	−2.02938800	−1.16778800
C	2.55562500	−0.63564600	−2.73733100
H	3.52381000	−0.33055700	−2.33525500
H	2.72109100	−1.41294000	−3.48479500
H	2.10853700	0.22880000	−3.23487900
C	0.28360000	−1.47958400	−2.13080700
H	−0.27961700	−0.61800100	−2.45547400
C	−0.12041200	−2.71541100	−2.62068200
H	0.55457800	−3.56101100	−2.51837300
C	−1.45978600	−2.97419700	−2.93595800
H	−1.75163400	−4.01937800	−2.97652400
C	−2.51890500	−2.07475500	−2.91322800
H	−3.48256200	−2.57602400	−2.88328900
C	−0.75957000	−1.30829300	−0.30519500
C	−2.13020600	−1.34804000	−0.58124000

H	−2.65907900	−0.42297400	−0.75963200
C	−2.94272100	−2.50521000	−0.28983000
C	−0.17770900	−2.50856600	0.35518400
C	−0.90354800	−3.80188100	0.27502100
C	−2.22100600	−3.77822500	0.00306900
O	−4.18526600	−2.48447000	−0.27169200
O	0.88415200	−2.44343400	0.96700100
C	−2.44809800	1.48172200	1.21005900
H	−3.47004800	1.14700500	1.00756300
H	−1.92687800	0.69148200	1.74842000
H	−2.51286600	2.36055200	1.85805900
C	−2.44691000	2.97606500	−0.83022600
H	−2.47886600	3.87899900	−0.21384600
H	−1.92907200	3.20868300	−1.76136600
H	−3.47947500	2.69949000	−1.06090300
C	−2.63859300	−0.65907800	−3.41398600
H	−1.73546400	−0.05905900	−3.37821900
H	−2.94729900	−0.72661500	−4.46332700
H	−3.43058800	−0.13948600	−2.87344000
C	−0.14362100	−5.04645800	0.61491100
H	0.19402400	−5.02340700	1.65311300
H	−0.76081000	−5.92532700	0.44436100
H	0.75582500	−5.11237500	−0.00194200
O	−2.95558000	−4.92873800	−0.04055400
C	−3.76732500	−5.15483200	1.12476500
H	−4.32803700	−6.06438500	0.92661400
H	−3.12397600	−5.29641900	1.99525100
H	−4.44546800	−4.31859000	1.27928000
H	−4.80910300	−0.73871500	−0.63554000
O	−4.90806700	0.20049200	−0.88236200
H	−5.81879100	0.29482200	−1.18055800
H	1.46755100	−3.60726000	3.85392700
O	1.80528300	−4.14589400	3.13094700
H	1.68322500	−3.59099500	2.34546800
H	−4.91731100	−3.82604200	−1.61111700
O	−4.69816400	−4.47676900	−2.29413700
H	−4.05691600	−5.03091500	−1.83054400

TS1xw
E(RM052X) = −1271.739294 a.u.
1 imaginary frequency −357.686 cm^{-1}

C	−0.08445500	−0.02274700	0.02133000
H	−0.12647700	0.03476000	1.10743300
C	1.42361000	−0.05888600	−0.37805100
H	1.59322400	0.70259200	−1.14568100
H	2.03690500	0.22377200	0.47933000
C	−0.73884900	1.20683800	−0.54740400
H	−0.91659800	1.19405900	−1.62149100
C	−1.06480800	2.31425500	0.12534300
C	1.86216800	−1.42171000	−0.93481000
H	1.99502400	−2.11652200	−0.10621200
C	3.19269000	−1.31859700	−1.69060500
H	3.96860300	−0.92646600	−1.03011900
H	3.51544500	−2.29379900	−2.05828000

H	3.10358500	−0.64514600	−2.54561600
C	0.73519700	−1.92656800	−1.79886600
H	0.48327000	−2.97657300	−1.74571100
C	0.29988400	−1.16384100	−2.87489800
H	0.83516200	−0.23745500	−3.06290900
C	−0.95078800	−1.27298100	−3.49099700
H	−1.28727100	−0.40441600	−4.04828900
C	−1.92741200	−2.20924200	−3.19892900
H	−2.92015800	−1.94547800	−3.54748100
C	−0.84750600	−1.30941900	−0.33491000
C	−2.09654800	−1.29288000	−0.96598100
H	−2.47891100	−0.37525900	−1.39099200
C	−3.11697300	−2.29710400	−0.72028000
C	−0.61830300	−2.42043300	0.62679700
C	−1.53145900	−3.60074200	0.65864900
C	−2.73836300	−3.50257400	0.07852500
O	−4.26961000	−2.19884400	−1.16028100
O	0.28326100	−2.38651100	1.45957200
C	−0.84523400	2.52473500	1.60220100
H	−1.80371700	2.52618100	2.13136300
H	−0.19310500	1.78097100	2.05781000
H	−0.39567400	3.50805800	1.76696100
C	−1.70071800	3.48312500	−0.58175400
H	−1.06131900	4.36868800	−0.51796800
H	−1.88993300	3.26441000	−1.63290200
H	−2.65174100	3.74456900	−0.10776400
C	−1.73075900	−3.67945500	−2.93169000
H	−1.22243500	−4.12439800	−3.79195100
H	−1.13261900	−3.90320700	−2.05152800
H	−2.69921600	−4.16817200	−2.82339300
C	−1.07963400	−4.78847100	1.44982300
H	−0.91751700	−4.50262000	2.49006100
H	−1.81567700	−5.58629500	1.39422000
H	−0.12187900	−5.15018100	1.07104300
O	−3.62522200	−4.53627500	0.10698800
C	−4.80659500	−4.30561200	0.89647700
H	−5.47148800	−5.13882900	0.68659800
H	−4.53894500	−4.29369400	1.95424000
H	−5.28086000	−3.37120700	0.60777400
H	1.22289600	−0.95394300	2.39871200
O	1.48415300	−0.16264500	2.89535300
H	1.18708600	−0.32931800	3.79600700
H	−4.97110400	0.52384300	−3.13827700
O	−4.17456400	−0.00464000	−3.02304200
H	−4.41459000	−0.70185400	−2.38921300
O	−5.06403100	−4.77351900	−2.36478400
H	−4.49800200	−5.18380500	−1.69980200
H	−5.06664100	−3.84876500	−2.07849700

TS2nw

E(RM052X) = −1271.746151 a.u.

1 imaginary frequency −364.055 cm^{-1}

C	−0.10118100	−0.04979300	0.06575800
H	−0.08579100	−0.09778000	1.15729900

C	1.35378200	−0.03592700	−0.45229300
H	1.36130000	0.34606800	−1.47104500
H	1.93908900	0.65298600	0.16009200
C	−0.86032600	1.17862900	−0.37277700
H	−1.20785500	1.16324000	−1.40195100
C	−1.12928200	2.26284000	0.35972000
C	1.96399400	−1.46715100	−0.43945000
H	2.72196200	−1.50535400	0.35113100
C	2.64671800	−1.82656700	−1.75801900
H	3.41052700	−1.08180300	−1.98951900
H	3.13546700	−2.80133000	−1.69792900
H	1.93082500	−1.83718400	−2.57974300
C	0.88503200	−2.42770500	−0.00112200
H	0.62578200	−2.29900600	1.03746100
C	0.57097400	−3.66823700	−0.55733700
H	0.98162100	−3.93172700	−1.52637900
C	−0.49263100	−4.42512300	−0.05657500
H	−0.93607000	−5.15412500	−0.72800900
C	−1.21358400	−4.15265100	1.10200100
H	−2.17441000	−4.65787500	1.13713700
C	−0.83682400	−1.31422800	−0.38615600
C	−1.84602200	−1.86063800	0.42080000
H	−1.95628600	−1.52122700	1.44408500
C	−2.99044100	−2.54345200	−0.12966100
C	−0.95935200	−1.51215300	−1.85808800
C	−1.95415600	−2.46941900	−2.39072800
C	−2.91896800	−2.92661200	−1.57297800
O	−4.01490100	−2.81371400	0.51666200
O	−0.27019900	−0.87669600	−2.65403000
C	−0.70861900	2.47680900	1.78959700
H	−1.58650500	2.62231700	2.42631300
H	−0.12372000	1.65470800	2.19804100
H	−0.10845800	3.38811000	1.86881700
C	−1.90622000	3.40849000	−0.23631000
H	−2.80926300	3.60681100	0.34917000
H	−1.31289700	4.32793800	−0.22501700
H	−2.20257100	3.19652200	−1.26376400
C	−0.69760900	−3.79012200	2.47158200
H	−1.34385600	−3.06958200	2.97378300
H	−0.74524500	−4.71139000	3.06177600
H	0.33557600	−3.45354000	2.49006000
C	−1.90267300	−2.76106300	−3.85894300
H	−2.18807300	−1.86476700	−4.41465900
H	−0.88607600	−3.02151900	−4.15694000
H	−2.57963400	−3.57448000	−4.10855900
O	−3.88956300	−3.76660200	−2.03441200
C	−5.14927800	−3.11778700	−2.28965900
H	−5.81798600	−3.89623500	−2.64678400
H	−5.53544900	−2.67684600	−1.37368900
H	−5.01598000	−2.35491200	−3.05835400
O	−2.30829600	0.65963400	−3.87516700
H	−1.45709600	0.23644300	−3.68806200
H	−2.10545500	1.37673200	−4.48502600
H	−3.95278800	−1.23960100	3.35837200
O	−3.45439400	−2.04845600	3.20088800
H	−3.79001200	−2.38230300	2.34874300

H	−3.91204100	−5.44470900	−0.80822700
O	−3.98208600	−5.68202800	0.12586800
H	−4.34978800	−4.87002100	0.50290400

TS2xw
E(RM052X) = −1271.743739 a.u.
1 imaginary frequency −353.411 cm^{-1}

C	0.05595200	0.03597400	−0.03123200
H	0.06815500	0.01968400	1.06088300
C	1.50512800	0.00091600	−0.53021700
H	1.51189400	0.12272500	−1.61151700
H	2.05408200	0.84246900	−0.10029800
C	−0.63817700	1.28296100	−0.52363800
H	−0.53124800	1.46135300	−1.59179300
C	−1.36301100	2.15262800	0.18627600
C	2.19638400	−1.33820700	−0.16193800
H	2.73793000	−1.18291200	0.77613700
C	3.19313200	−1.75251400	−1.24552000
H	3.92033000	−0.95771900	−1.42380100
H	3.73370700	−2.65825000	−0.96408200
H	2.65988700	−1.94242000	−2.17816000
C	1.15767600	−2.40024800	0.10213800
H	0.91094900	−3.06871100	−0.71161900
C	0.84599800	−2.69891700	1.42302400
H	1.42131400	−2.17644100	2.18289000
C	−0.32608800	−3.31410400	1.86572100
H	−0.59304900	−3.17264500	2.90707900
C	−1.33232700	−3.80067800	1.03788700
H	−2.27224700	−4.01084600	1.53599800
C	−0.75407700	−1.19805800	−0.44755800
C	−1.79432200	−1.64371100	0.37450300
H	−1.86168000	−1.26948000	1.38868000
C	−3.07040300	−2.10645300	−0.15338900
C	−0.88527800	−1.48046100	−1.89617500
C	−2.11886100	−2.14662600	−2.43070500
C	−3.15313000	−2.39533400	−1.61111700
O	−4.06722000	−2.26804400	0.56040100
O	−0.02433900	−1.15210000	−2.71193600
C	−1.64206800	2.05590000	1.66295500
H	−1.33284400	2.97618400	2.16713300
H	−2.71766400	1.94877100	1.83312400
H	−1.14426100	1.21849700	2.14695800
C	−1.99750900	3.34660900	−0.48004200
H	−1.79163200	3.36771300	−1.55034100
H	−3.08219400	3.33784600	−0.33542900
H	−1.62724100	4.27679200	−0.03862700
C	−1.11065600	−4.54461900	−0.25127000
H	−0.32790300	−5.29366400	−0.10481500
H	−2.03394700	−5.04610800	−0.53649500
H	−0.78355600	−3.92278400	−1.08343100
C	−2.12462300	−2.48564900	−3.88798900
H	−1.37221900	−3.25316000	−4.08407900
H	−3.10050500	−2.85903000	−4.18748700
H	−1.85882300	−1.60851000	−4.47856900

O	−4.28992200	−3.00372300	−2.06056100
C	−5.43607800	−2.14023900	−2.12935400
H	−6.25788700	−2.76179500	−2.47474500
H	−5.66176200	−1.72854800	−1.14830600
H	−5.24855900	−1.33996400	−2.84774100
H	1.73488600	−4.03922700	−3.40349200
O	0.85383400	−3.79174400	−3.10300100
H	0.76624800	−2.84476400	−3.28947700
H	−3.73283800	−2.73063000	3.87736100
O	−3.12817900	−2.29544800	3.26747300
H	−3.61286600	−2.23095400	2.42727400
O	−4.46651500	−5.08285100	−0.03312800
H	−4.49729400	−4.83872600	−0.96718200
H	−4.62260500	−4.23107700	0.39713500
